# IMPLEMENTATION SCIENCE: A STRATEGY FOR ADDRESSING HEALTH EQUITY AMONG RACIALLY DIVERSE OLDER ADULTS

**DOI:** 10.1093/geroni/igad104.1760

**Published:** 2023-12-21

**Authors:** Karen Bullock, Barbara Mendez Campos

**Affiliations:** Boston College, Boston, Massachusetts, United States; Boston College, Boston, Massachusetts, United States

## Abstract

**Objective:**

Implementation science as a strategy for helping diverse populations of older adults address barriers to mental treatment, has become increasingly important since COVID19. This paper disseminates information about the results of an interdisciplinary intervention that tailored mental health services to racially and ethnically diverse community-dwelling older adults. Method: An interdisciplinary team of researchers (MD, APRN, MSW) performed an intervention that consisted of weekly visits to the community and provided diagnostic, triage, and psychosocial services to older adults. Evaluation of the intervention included data collected at baseline and three-month follow-up visits.

**Results:**

350 elderly residents received mental health screening with some assessment of physical health and well-being. The number of visits per resident during the intervention phase, ranged from 1 to 11, with an average of 3. Tailoring the intervention to the specific cultural needs of the participants led to the development of a weekly community engagement group for monolingual Spanish-speaking residents and others. 49% of those receiving the intervention, self-reported their race and/or ethnicity as African American or Caribbean, 43% were Latino, and 8% were Caucasian, all of whom self-referred as participants in this health service outreach program. Initial evaluations resulted in referral to community providers (60%), team case management (20%), or no services needed (20%).

**Conclusion:**

Research studies designed to have positive impacts on older adults that have been historically underrepresented in health services research may improve its effectiveness by tailoring processes to be community informed and culturally responsive to structural and system barriers to treatment.

